# Development of engineered magnetic liposome/exosome hybrid as a novel caffeine nanocarrier for restraining liver fibrosis induced in rats

**DOI:** 10.1038/s41598-025-31169-w

**Published:** 2026-02-06

**Authors:** Yara E. Elakkad, Hanan Refai, Hanaa H. Ahmed, Ahmed N. Abdallah, Menna M. Abdellatif, Ola A. M. Mohawed, Rehab S. Abohashem

**Affiliations:** 1 Pharmaceutics Department, College of Pharmaceutical Sciences and Drug Manufacturing, Misr University for Science and Technology, 6th of October City, Giza 12566 Egypt; 2 Department of Pharmaceutics, College of Pharmacy, Alfarahidi University, Baghdad, Iraq; 3 Hormones Department, Medical Research and Clinical Studies Institute, National Research Centre, Giza, Egypt; 4 Stem Cell Lab, Center of Excellence for Advanced Sciences, National Research Centre, Giza, Egypt; 5 Industrial Pharmacy Department, College of Pharmaceutical Sciences and Drug Manufacturing, Misr University for Science and Technology, 6th October City, Giza 12566 Egypt

**Keywords:** Liver fibrosis, Thioacetamide, Caffeine, Nanoliposomes, Exosomes, SPION, Exosome/liposome hybrid, Biochemistry, Stem cells

## Abstract

Liver fibrosis, a severe health issue linked to liver injury, inflammation, and cell death, often progresses to liver cancer without effective treatments. In this study, an exosome-nanoliposome hybrid loaded with caffeine (Caff) was developed for the management of liver fibrosis. The role of superparamagnetic iron oxide nanoparticles (SPION) in promoting targeted delivery to the liver was also investigated. Caff-loaded liposomes with variable phospholipid and stearylamine molar ratios were prepared and characterized. The formula with optimized characteristics (Caff/liposomes) selected by Design Expert exhibited a particle size of 179.73 ± 3.1 nm, a polydispersity index of 0.19 ± 0.02, an entrapment efficiency of 89.79 ± 21%, and a zeta potential of -35.50 ± 1.1 mV, and was used to encapsulate SPION. Caff/SPION liposomes were further loaded into exosomes, isolated from bone marrow-mesenchymal stem cells, and visualized by TEM. The biological experiment was conducted on rats with thioacetamide-induced liver injury to reveal the efficacy of exosome-Caff/SPION liposome hybrid (F1) in comparison to exosome-Caff/liposomes (F2) and exosome-SPION/liposomes (F3). Among all the tested formulations, F1 demonstrated the most promising results regarding liver function markers (ALT and AST), inflammatory molecules (IL-6, IL-10, TLR-4, Myd88, NF-κB), fibrotic protein (α-SMA), hepatocyte proliferative capacity (PCNA), as well as liver histoarchitecture. This finding suggests that the engineered Caff/SPION liposome-exosome hybrid can effectively enhance the targeting of the drug and the homing of exosomes to the injured liver, thereby representing a novel strategy for treating liver fibrosis.

**Supplementary Information:**

The online version contains supplementary material available at 10.1038/s41598-025-31169-w.

## Introduction

 Chronic liver diseases are considered a primary cause of death for about 2 million people each year, accounting for 4% of all deaths worldwide (or 1 in every 25 deaths worldwide). This highlights the substantial health burden of chronic liver disorders worldwide^1^. The primary sources of chronic liver diseases include autoimmune and genetic disorders, alcoholic and nonalcoholic steatohepatitis, and viral-related liver diseases^2^. Liver damage triggers inflammatory responses, with chronic inflammation activating hepatic stellate cells. These cells undergo transdifferentiation into myofibroblasts, the liver’s primary extracellular matrix producers. Over time, excessive matrix production results in liver fibrosis^3^.

Treating liver fibrosis aims to stop the fibrotic process while restoring liver function. Targeting the underlying cause of liver damage is the most successful strategy. Therefore, the development of cutting-edge therapeutic approaches for liver fibrosis has garnered growing attention in recent years. Inhibitors of transforming growth factor-beta (TGF-β) signaling or collagen synthesis are examples of antifibrotic medications that specifically target fibrogenesis-related biological pathways. Additionally, early clinical research on cell therapy, such as the implantation of mesenchymal stem cells or hepatocytes, has yielded promising outcomes^4^. MSCs possess immunomodulatory properties that can help reduce inflammation and fibrosis in the liver. Furthermore, MSCs can differentiate into hepatocytes and promote tissue regeneration^5^.

Exosomes, small extracellular vesicles derived from stem cells, have garnered considerable interest as a potential alternative to stem cells in various therapeutic applications, including the treatment of liver fibrosis^6,7^. These vesicles are rich in biologically active substances, including growth factors, cytokines, and microRNAs, which can influence cellular behaviour and promote tissue regeneration. Their compact size, stability, and capacity to traverse biological barriers make them a promising option for targeted therapies^8^. Exosome-based therapies can avoid certain limitations associated with stem cell therapy. These challenges encompass problems such as necrosis or abnormal differentiation due to stress responses and immune rejection related to cell transplantation.

Exosome-based therapies are considered safer and do not carry the potential risk of tumor formation associated with stem cells^9^. Another advantage of using exosomes is their potential to serve as an off-the-shelf therapy. Exosomes can be easily gathered and preserved for later use, unlike stem cells, which need to be isolated, expanded, and possibly immunomodulated before delivery. This property of exosomes shortens the time and lowers the cost required for the production process in cell-based medicines^10^. Exosomes also exhibit immunomodulatory qualities comparable to stem cell characteristics^11^. Exosomes can inhibit inflammation and modulate the immune system, playing a pivotal role in addressing the underlying causes of liver fibrosis. They regulate immune cell activation and boost the generation of regulatory T cells, thereby slowing the progression of fibrosis^12^. A recent study demonstrated that MSC-derived exosomes significantly reduce collagen deposition, inhibit the epithelial-to-mesenchymal transition (EMT) process, improve liver function, and decrease inflammation in a mouse model of liver cirrhosis^13^.

Liposomes are commonly utilized as drug delivery systems because they can self-assemble, encapsulate water-soluble and lipophilic agents, and provide superior pharmacokinetic profiles. However, one drawback of liposomes is their vulnerability to elimination by the mononuclear phagocyte system (MPS), which limits their effectiveness in transporting drugs. Recent research has shown that hybrid nanoparticles, formed by combining engineered exosomes and liposomes, improve drug delivery and protect against MPS clearance. These hybrid nanovesicles demonstrate excellent biocompatibility, prolonged circulation time, and resistance to removal by the MPS^14^.

Superparamagnetic iron oxide nanoparticles (SPIONs) have demonstrated significant potential in various biomedical applications for targeting and guiding exosomes, as well as different drug delivery systems. Labeling nanocarriers with SPIONs can attract and guide them to specific tissues or cells using an external magnetic field. This targeted delivery method enables the consistency of carriers at the intended site, thereby enhancing therapeutic effectiveness and minimizing off-target effects^15^.

Lately, attention to utilizing caffeine as a potential treatment for liver fibrosis has increased because of its positive impact on liver health. Caffeine, a psychoactive substance found in popular beverages such as coffee and tea, is well-known for its stimulating properties and has been extensively researched for its effects on various organs, including the liver. Numerous studies have indicated that caffeine might have protective properties against liver fibrosis^16^. A possible explanation is that caffeine can inhibit hepatic stellate cell activity and reduce collagen generation, potentially slowing the progression of liver fibrosis^17^. The anti-inflammatory characteristics of caffeine have been reported, which can be advantageous in liver fibrosis. Chronic inflammation is a significant factor in initiating and advancing fibrotic liver conditions^18^. Additionally, a recent investigation revealed that caffeine can decrease the expression of alpha-smooth muscle actin (α-SMA), connective tissue growth factor (CTGF), and transforming growth factor-beta (TGF-β), while suppressing mitogen-activated protein kinases (MAPKs) and the phosphorylation of Smad3, thereby activating Smad3^19^.

The objective of the current study was to formulate hybrid exosomes-liposomes nanoparticles loaded with caffeine to inhibit liver fibrosis induced in rats. The purpose of this research was further expanded to explore the role of SPION in enhancing the targeting and homing of the hybrid exosomes-liposomes to the liver.

## Materials and methods

### Materials

Caffeine, soy phosphatidylcholine, Bovine serum albumin (BSA), phosphate-buffered saline (PBS), Serum-free medium 199 with HEPES 25 mM, antibiotics like streptomycin and penicillin, fetal bovine serum (FBS), 0.25% trypsin/EDTA, and Dulbecco’s Modified Eagle’s Medium (DMEM) were purchased from Sigma-Aldrich (USA). SPION (Magnetite (Fe_3_O_4_, iron IV oxide nanoparticles with an average particle size of 25 ± 5 nm) was obtained from Nanotech Company (Cairo, Egypt). We bought disodium hydrogen phosphate, potassium dihydrogen phosphate, and sodium chloride from El-Nasr Pharmaceutical Co. (Cairo, Egypt). All other chemicals met the quality criteria as per international standards.

### Experimental setup

Using Design Expert software (version 13; Stat-Ease Inc., MN, USA) to construct the runs of 2^3^ modified fractional two-level factorial design. The studied factors were phospholipid molar ratio, stearylamine molar ratio (X_2_), and sonication time (X_3_). All the formulae, as shown in Table 1, were assessed through measurement of entrapment efficacy (EE, Y_1_, %), particle size (PS, Y_2_, nm), and zeta potential (ZP, Y_3_, mV).

**Table 1:** Experimental runs, independent and dependent variables of caffeine-loaded liposomes (Caff/liposomes) following a 2^3^ modified fractional factorial design.

Preparation Code	Phospholipid molar ratio	Stearylamine molar ratio	Sonication time (min)	EE % (Y_1_)	PS (nm) (Y_2_)	PDI (Y_3_)	ZP (mV) (Y_4_)
P1	15	15	10	94.35 ± 1.9	1598 ± 12.4	0.60 ± 0.005	−5.69 ± 1.8
P2	10	7.5	15	86.73 ± 1.1	267.49 ± 3.9	0.44 ± 0.07	−31.30 ± 2.3
P3	10	0	10	84.97 ± 1.0	208.41 ± 2.2	0.21 ± 0.03	−43.90 ± 1.6
P4	12.5	0	5	87.89 ± 1.2	202.80 ± 5.15	0.21 ± 0.06	−41.62 ± 1.4
P5	12.5	0	15	89.79 ± 2.1	179.73 ± 3.1	0.19 ± 0.02	−35.50 ± 1.1
P6	12.5	7.5	10	89.65 ± 1.5	414.0 ± 2.3	0.62 ± 0.03	−32.50 ± 0.9
P7	15	15	5	89.52 ± 0.9	1511.0 ± 6.9	0.73 ± 0.04	−11.0 ± 1.4
P8	12.5	7.5	10	89.65 ± 1.1	536.90 ± 4.7	0.69 ± 0.05	−38.20 ± 1.9
P9	15	15	15	89.21 ± 1.8	1504.0 ± 7.9	0.56 ± 0.07	−4.66 ± 0.8
P10	12.5	1.5	10	88.60 ± 1.3	287.23 ± 4.6	0.32 ± 0.04	−51.0 ± 1.2

### Formulation of caffeine-loaded liposomes (Caff-liposomes)

A thin-film hydration method was used to formulate liposomes, in which the appropriate amounts of soy phosphatidylcholine and a cationic surfactant were dissolved in a methanol/chloroform mixture (2:1 v/v). The organic solvent system was removed gradually using a rotary evaporator at 40 °C under reduced pressure. So, a very thin film of dry lipid was formed on the inner surface of the flask^20^. The dried lipid film was progressively hydrated with 10 mL of 0.3% caffeine aqueous solution.

### Characterization of caffeine-loaded liposomes (Caff-liposomes)

#### Determination of the entrapment efficiency of caffeine

One mL from each formula was centrifuged at 20,000 rpm for 1 h at 4 °C using a cooling centrifuge (Sigma 3 K 30, Germany). After centrifugation, methanol was used to lyse the sediment, which was analyzed using a UV-Vis spectrophotometer (Shimadzu UV-1650, Japan). The EE% was calculated using this equation^21^: 

1 $$\:\mathrm{EE}{\%}\:=\frac{\text{Amount of encapsulated drug present}}{\text{Total amount of drug present}}\times\:\:100\:$$

#### Determination of the particle size (PS), polydispersity index (PDI), and zeta potential (ZP) of Caff-liposomes

PS was measured by dynamic light scattering (DLS) using a Zetasizer (Zetasizer Nano ZS, Malvern Instruments, Worcestershire, UK). The same instrument was used to determine PDI and ZP. The measurements were performed at 25 °C after sample dilution (1:100) with deionised water. The measurements were done in triplicate.

### Selection of the optimized formula

The optimized formula was selected using Design-Expert software. The desirability tool selected the formula with the lowest PDI and PS and the highest EE% and ZP.

### In vitro drug release study

The in vitro release of caffeine from optimized caffeine-loaded liposomes was conducted using a USP dissolution testing apparatus type II (Hanson Research Corp, CA, USA). The dissolution media consisted of 50 mL of PBS (pH 7.4), and 1 mL of the formula (containing 3000 µg of caffeine) was instilled into two open-ended tubes. One end of each tube was sealed with a cellulose membrane, and the other end was fixed to the shaft of the dissolution device. The caffeine release profile from its equivalent aqueous solution was also performed. The dissolution media temperature was maintained at 37 ± 1 °C, and the shaft was rotated at 100 rpm. At definite time intervals (1, 2, 3, 4, 5, 6, 7, and 8 h), 1 mL samples were taken from the dissolution media and substituted with an equal volume of fresh media. The concentration of caffeine was determined spectrophotometrically at λmax 272 nm. The percent of drug released after 8 h (Q_8%_) was estimated. The release data was kinetically assessed employing diverse release models (Higuchi, first-order, Peppas, Hixson-Crowell, and zero-order).

### Encapsulation of SPION into the optimized caffeine-loaded liposomes (Caff/SPION liposomes)

 The caffeine-loaded liposomal preparation with optimal characteristics (P5) was loaded with SPION using the same method described in the section “Formulation of caffeine-loaded liposomes (Caff-liposomes)”, except that SPION (2 mg/mL) was added to the organic phase. Before the FTIR analysis, the formula was vacuum-dried.

### Determination of loading efficiency of SPION

The exact quantity of SPION loaded into the caffeine-loaded liposomal preparation (P5) was estimated using potassium thiocyanate. One milliliter of Caff/SPION liposomes was separated at 20,000 rpm for 1 h at 4 °C using the cooling centrifuge. First, 0.1 mL of supernatant was diluted with 2 mL of 3% 6 N HCl containing 1% H_2_O_2_. Then, 2 mL of 5% potassium thiocyanate solution was added^22^. The SPION concentration was determined by measuring absorbance at 477 nm. The loading efficiency was calculated using the following equation^23^:

2 $$\text{Loading efficiency}\:=\frac{Weight\:of\:SPION\:encapsulated}{Weight\:of\:lipid}$$

Utilizing the loading efficiency calculated together with the hydrodynamic diameter of the liposome formula, the number of SPION encapsulated in each liposome could be calculated using the following equation^23^:

3 $$\mathrm{N}=\:\frac{NS}{NL}$$

Where N: number of SPION encapsulated in each liposome, NS: total number of encapsulated SPION, and NL: total number of liposomal vesicles.

NS was calculated by dividing the total mass of encapsulated SPION by its density (5.1 g/cm^3^) and the volume of a single SPION with an average diameter of 25 nm (8.181 × 10^− 18^cm^3^). The NL was calculated from the average liposomal radius, allowing the total area to be determined. Dividing the total area by the cross-sectional area of soy phosphatidylcholine (*a*0 = 0.72 nm²) and then multiplying the lipid concentration of the formula by the Avogadro constant, the number of total lipid molecules per unit volume can be obtained.

### Fourier-transform IR spectroscopy (FT-IR)

FTIR spectroscopy was used to assess the compatibility between the drug and excipient. FTIR-8400 (Shimadzu, Kyoto, Japan) was used for IR spectra analysis of caffeine, SPION, caffeine-SPION physical mixture, and the dried Caff/SPION-liposomes at ambient temperature. A disk containing approximately 2–3 mg of each sample was compressed using dry potassium bromide (KBr). Then, it was subsequently scanned at 400–4000 cm^-1^^24^.

### Culturing of bone marrow mesenchymal stem cells (BM-MSCs)

MSCs were isolated from the BM of rats and then incubated at 37 °C in 5% CO_2_ using DMEM media provided with 10% FBS and 1% antibiotic. DMEM media were replaced continuously to eliminate non-specific cells and allow the cells to expand until they reached confluence. The adherent cells were washed with PBS, and fresh media were added every 3–4 days. The initial adherent, spindle-shaped cells appeared as separate cells under inverted microscopy on the 3rd day. The culture became more confluent and reached 65–70% confluence within 4–8 days. Then, cultures were washed twice with PBS. Then, cells were trypsinized with 0.25% Trypsin-EDTA (GIBCO-USA) for 3 min at 37 °C. A complete media was added to stop trypsin action. Then, the sample was centrifuged for 10 min at 200xg (2000 rpm). Cell pellets were suspended in complete medium, and the resulting cultures were designated as 1 st passage cultures. The last step was repeated until the 4th passage was completed^25^.

### Characterization of BM-MSCs

MSCs were identified by their adherence to the culture flask and by their characteristic spindle-shaped morphology, as observed under inverted microscopy. MSCs-specific surface antigens (CD90, CD73, and CD105) were analyzed by flow cytometry. After that, the cells were washed and suspended in PBS supplemented with 3% FBS, consisting of a saturating concentration (1:100) dilution of the following fluorescein isothiocyanate-conjugated monoclonal antibodies: anti-CD90, anti-CD73, and anti-CD105 (BD Pharmingen, USA). Forward scatter analysis (Becton Dickinson, Canada) was used to investigate the specimens^26^.

### Exosomes isolation

Exosomes were isolated from the 4th passage supernatants of MSCs (5 × 10^6^ cells/ml) and cultivated in FBS-free RPMI media with 0.5% BSA (Sigma, USA). Two days after incubation, the media were centrifuged at 10,000 rpm for 10 min to remove any cell debris; then, the supernatants were collected and filtered through a 0.22 μm syringe filter. Then, cell-free supernatants were subjected to 1 h ultracentrifugation at 100,000xg (Beckman Coulter Optima L-90 K ultracentrifuge) at 4 °C to collect the exosome pellet. Then, the pellets were resuspended in 200 µL PBS and stored at −80 °C for long-term conservation^27^.

### Determination of the total protein content of exosomes

The BCA protein assay kit (Thermo Scientific Pierce, Rockford, IL, USA) was used to determine the protein content of the collected exosome sample. The exosome sample (25 µl) was transferred to 96-well plates, to which 200 µl of working reagent was added. Then, the plate was incubated for 30 min at 37 °C before being analyzed spectrophotometrically at 562 nm (Victorz, Perkin-Elmer, Waltham, MA, USA). The identical process was used to create the standard curve for various bovine serum albumin (BSA) concentrations (50–250 µg/ml)^28^.

### Loading of Caff/SPION liposomes into exosomes (liposome/exosome hybrid)

According to Farouk et al. (2024), the loading of Caff-SPION liposomes into exosomes was carried out using the probe sonication method with some modifications^29^. The liposome/exosome hybrid ratio was 1:1. In summary, the mixture was subjected to six cycles of a 4-second pulse and a 2-second pause (750 volts, 20% power) under cooling on ice for 2 min, followed by a single sonication. After that, the sample was incubated at 37 °C for one hour without shaking.

### Measuring the entrapment efficiency of caffeine into exosomes

Entrapment efficiency was evaluated using a dialysis tubing cellulose membrane (Sigma-Aldrich, Massachusetts, USA) against PBS by placing 2 mL of exosomes/liposomes hybrid in a cellulose dialysis bag, which was cut out with scissors. The dialysis bag was then sealed correctly from both the top and bottom, placed in PBS (pH 7.4), and gently shaken using a shaking incubator at 50 rpm at room temperature. The media were gathered after an hour and replaced with fresh PBS for an additional 1 h. Subsequently, the collected media underwent syringe filtration and were analyzed by spectrophotometry at 273 nm to determine the entrapment efficiency. The EE% was determined following this equation^30^:

4 $$\:\mathrm{EE}{\%}\:=\frac{\text{Total amount of drug} -\text{amount of unloaded drug}}{\text{Total amount of drug}\:}\times\:\:100$$

### Morphology and PS measurement

The optimized Caff/SPION liposome (P5), exosomes, and exosome-Caff/SPION liposome hybrid (F1) were analyzed for morphology and PS using TEM. A small amount of liposomal suspension was pulled into the copper grid surface and left for 10 min. Excess liquid was blotted out using filter paper. Then, the phosphotungstic acid stain was inserted dropwise for 10 min. The filter paper was used to remove excess liquid, and TEM was used to investigate it. The transmitting beam of electrons passes through the thin specimen to create a 2D image of liposomes, using an accelerating voltage of 20–30 kV and a working distance of 15–25 mm. The system (Jeol T300, Japan) was used for digital capture and analysis^31^. The PS of the exosome-liposome hybrid was also evaluated via DLS, as described in the section “Determination of the particle size (PS), polydispersity index (PDI), and zeta potential (ZP) of Caff-liposomes”, with assessment of PDI and ZP.

### Stability testing

To assess the impact of storage on the optimized exosome-Caff/SPION liposome hybrid (F1), a sample was maintained in a tightly sealed glass vial at 4 °C for 7 months. The developed system was visualized by TEM at the end of the storage duration. Furthermore, the PS, ZP, and PDI were assessed as described in the section “Determination of the particle size (PS), polydispersity index (PDI), and zeta potential (ZP) of Caff-liposomes”, and the EE% was examined as described in the section “Measuring the entrapment efficiency of caffeine into exosomes”.

### Biological experimentation

This study was conducted on adult female *Wistar* rats (weighing 180–200 g) acquired from the National Research Centre (Egypt). Animals stayed in a standardized environment (12-h light and dark cycle) and were permitted food and tap water *ad libitum*. Before the experiment began, the Animals were kept for 15 days for acclimatization.

#### Ethical approval

The experimental protocol was conducted in accordance with the guidelines of the National Institutes of Health (NIH) and with the approval of the Institutional Medical Research Ethics Committee, National Research Centre, Giza, Egypt (Registration No. 62311122022). The study is reported in accordance with the ARRIVE guidelines.

#### Experimental groups

After the adaptation, the rats were grouped into six groups (8 rats/group); Group 1 was the control group, in which the rats were injected intraperitoneally with 1 ml of PBS three times weekly for 6 weeks. Group 2: Control + F1 (Exosome-Caff/SPION liposome hybrid) in which the rats were administered with 1 ml of the hybrid intravenously on days 3, 6, 9, and 12^32,33^. Group 3: Thioacetamide (TAA) group in which the rats were administered TAA intraperitoneally (100 mg/kg) 3 times/week for 6 weeks to induce liver fibrosis^34^. Group 4: TAA + F2 (Exosome-Caff/liposomes hybrid in which the rats were given TAA for 6 weeks, after which 1 mL of the hybrid was injected intravenously on days 3, 6, 9, and 12^32^. Group 5: TAA + F3 (Exosome-SPION/liposome hybrid in which the rats were given TAA for 6 weeks, after which 1 ml of the hybrid was administered intravenously on days 3, 6, 9, and 12^32^. Group 6: TAA + F1 (Exosome-Caff/SPION liposome hybrid) in which the rats were given TAA for 6 weeks, after which 1 ml of the hybrid was injected intravenously on days 3, 6, 9, and 12^32^. For the enhanced magnetic targeting of SPION-loaded formulae, a magnet bar (2 cm) was to be affixed over the liver region using surgical adhesive tape. The total duration of the experiment was 68 days, from the day of administration of TAA until the day of animal sacrifice.

#### Biochemical analysis

Two weeks after the last day of treatment, the rats were fasted overnight then sacrificed by cervical dislocation under full anesthesia by intraperitoneal injection of ketamine (90 mg/kg) and xylazine (5 mg/kg)^35^. Then, blood specimens were collected from the tail vein. The collected blood specimens were centrifuged under cooling (at 4 °C) for 15 min at 3000 rpm to obtain sera. Samples were then collected and stored at −20 °C for the estimation of alanine aminotransferase (ALT), aspartate aminotransferase (AST), interleukin-6 (IL-6), and IL-10. After the blood samples were taken, the rats were euthanized by cervical dislocation, and their livers were excised and portioned into three fractions. The first portion was weighed and homogenized in ice-cold PBS (pH 7.4). The resulting suspensions (10% w/v) were then centrifuged for 15 min at 4 °C and 3000 rpm to obtain the supernatants, which were collected and stored at −20 °C for further biochemical analysis of alpha-smooth muscle actin (α-SMA) and proliferating cell nuclear antigen (PCNA). A 2nd fraction was frozen in liquid nitrogen and immediately stored at − 80 °C for RNA extraction. A 3rd fraction was fixed in 10% formalin saline for histological procedures.

#### Colorimetric assay

According to the manufacturer’s instructions, liver function was assessed by measuring serum levels of ALT and AST using spectrophotometric kits (Biodiagnostic, Cat# AL1031 and AS1061, respectively).

#### ELISA assay

IL-6 (Cloud-Clone, Cat# SEA079Ra) and IL-10 (Cloud-Clone, Cat# SEA056Ra) levels in serum were quantified through ELISA kits. Hepatic contents of α-SMA (MyBioSource, Cat# MBS266620) and PCNA (FineTest, Cat# ER1234) were assayed by ELISA kits according to the manufacturer’s protocols.

#### Gene expression analysis

Total RNA was extracted from the liver tissues using the RNeasy Mini Kit from Norgen, Canada (Cat# 55200). One microgram of total RNA was reverse-transcribed using a reverse transcription kit from Norgen, Canada (Cat# 54420). qPCR was performed according to the manufacturer’s instructions using a HERA SYBR Green PCR kit (Willowfort, UK, Cat# WF10308001). Specific primers for nuclear factor kappa B (NF-κB)^36^, myeloid differentiation primary response 88 (Myd88)^31^, toll-like receptor 4 (TLR-4)^37^, and housekeeping gene, glyceraldehyde 3-phosphate dehydrogenase (GAPDH), were used for qPCR^38^. PCR cycling was performed as follows: initial denaturation at 94 °C for 15 min, followed by 40 cycles of denaturation at 94 °C for 15 s, annealing at 60 °C for 30 s, and extension at 72 °C for 30 s, all for 5 min. The primer sequences for each gene are listed in Table 2. The main equations used were: ∆Ct = Ct (gene of interest) – Ct (housekeeping gene), followed by ∆∆Ct = ∆Ct (treated sample) – ∆Ct (untreated sample). The overall formula was 2^–∆∆Ct^ to calculate the relative fold of change in gene expression.

**Table 2:** Primer sequences of the target genes for RT-qPCR.

Gene name	Forward primer	Reverse primer
NF-κB (Al-Rasheed et al., 2016)	CATGAAGAGAAGACACTGACCATGGAAA	TGGATAGAGGCTAAGTGT AGACACG
TLR-4 (Zhou et al., 2018)	CGCTCTGGCATCATCTTCAT	CTCCTCAGGTCAAAGTTGTTGC
Myd88 (Zhou et al., 2018)	GAGATCCGCGAGTTTGAGAC	TTGTCTGTGGGACACTGCTC
GAPDH (Wu et al., 2007)	CACCCTGTTGCTGTAGCCATATTC	GACATCAAGAAGGTGGTGAAGCAG

#### Histopathological procedures

Following a 24-hour fixation of liver tissues in formalin saline (10%), the tissues were washed in tap water and serially diluted with ethyl alcohol (70%, 80%, 90%, and 100%) for dehydration. Then, samples were cleared in xylene and embedded in paraffin beeswax at 56 °C in a hot air oven for 24 h. Paraffin bee wax tissue blocks were segmented at a thickness of 4 microns by rotary microtome. After routine dewaxing and hydration, H&E was applied, and an optical microscope was used to detect the histological changes^39^.

#### Toxicity study

To evaluate the potential effects of F1 (Exosome–Caffeine/SPION liposome hybrid) on vital organs other than the liver, the kidney and spleen tissues of Group 2 healthy rats were examined histopathologically, as described in the section “Histopathological procedures”.

### Statistical analysis

Results were expressed as Mean ± standard deviation. Moreover, mean comparisons and analysis of variance (ANOVA) were used, and *p* < 0.05 was considered statistically significant. All statistics were calculated using SPSS software (Chicago, Illinois, USA, Version 20).

## Results

### Factorial design optimization

During the initial screening to investigate the independent variables that could influence the features of caffeine-loaded liposome formulae, it was found that the phospholipid and stearylamine molar ratio, as well as the sonication time, affected the features of the liposomes; therefore, these factors were identified as independent variables. A modified fractional two-level factorial design with two center points was selected for this study to investigate multiple formulation factors. This approach substantially reduces experimental time, materials, and costs compared to full factorial designs, while preserving the ability to estimate main effects and the most relevant low-order interactions for optimization, assuming higher-order interactions are negligible. The inclusion of center points enabled the detection of nonlinearity and improved model reliability. A modified fractional factorial design was employed, in which the levels of stearylamine were adjusted across selected experimental runs (F10) to span a broader concentration range. This approach enabled evaluation of both typical and off-center levels of stearylamine, thereby enhancing the investigation of its effects on formulation properties and facilitating a more comprehensive optimization process.

The developed factorial models were validated by comparing predicted values with experimentally observed results using diagnostic plots (Supplementary Figures S1-S4). Additionally, adjusted and predicted R² values were within 0.2 of each other, indicating good predictive power and model robustness. Adequate precision ratios exceeding 4 further demonstrated that the model’s signal-to-noise ratio was sufficient for reliable optimization. The design output is shown in Tables 3 and 4.

**Table 3:** The *p* and F values of the 2^3^ modified fractional factorial design analysis.

Source	EE (%)	PS (nm)	PDI	ZP (mV)
Model *p*-value	< 0.0004	0.0002	0.0250	0.0057
Model F-value	34.17	41.40	6.60	12.23
X_1_ = A; Phospholipid Molar ratio (*p*-value)	< 0.0002	0.2472	0.8677	0.5216
X_1_ = A; Phospholipid Molar ratio (F-value)	66.17	1.64	0.030	0.463
X_2_ = B; Stearylamine Molar ratio(*p*-value)	0.152	< 0.0001	0.0073	0.0024
X_2_ = B; Stearylamine Molar ratio (F-value)	2.68	84.52	15.86	25.16
X_3_ = C; Sonication time (*p*-value)	0.141	0.316	0.451	0.501
X_3_ = C; Sonication time (F-value)	2.68	1.19	0.649	0.511
Significant factors	X_1_	X_2_	X_2_	X_2_

*EE* Entrapment efficiency, *PS* Particle size, *PDI* Polydispersity index, *ZP* Zeta potential.

**Table 4:** The adequate precision and R^2^ values of the 2^3^ modified fractional factorial design analysis.

Source	EE (%)	PS (nm)	PDI	ZP (mV)
Adequate precision	19.75	15.49	6.71	8.41
R^2^	0.944	0.953	0.767	0.859
Adjusted R^2^	0.917	0.930	0.651	0.789
Predicted R^2^	0.805	0.855	0.534	0.654

*EE* Entrapment efficiency, *PS* Particle size, *PDI* Polydispersity index, *ZP* Zeta potential.

### Characterization of Caff/liposomes

#### Drug entrapment efficiency

**Fig. 1:**
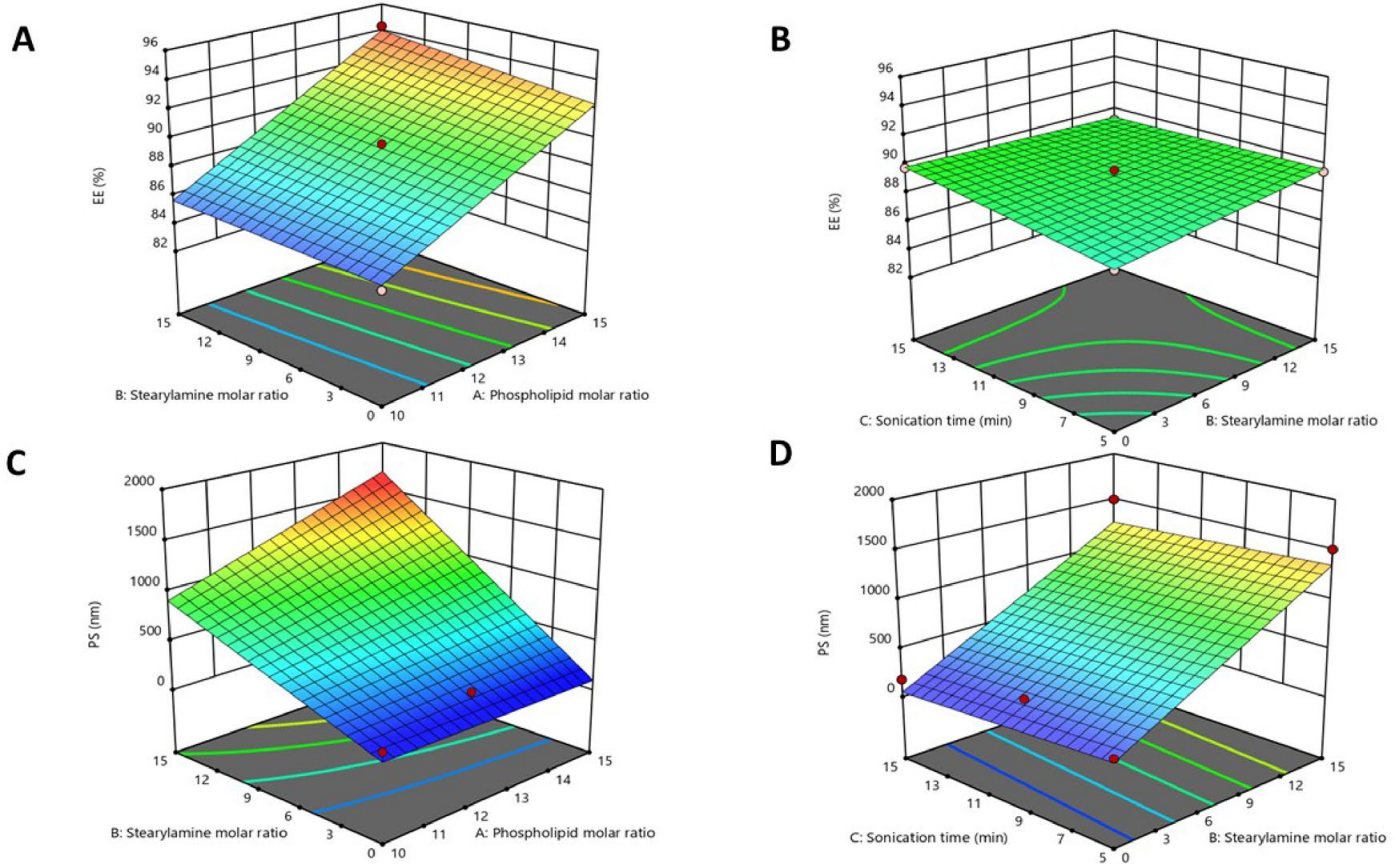
(A-B). Three–dimensional response surface plots for the effect of phospholipid molar ratio (X1), stearylaminemolar ratio (X2), and sonication time (X3) on EE%. (C-D). Three–dimensional response surface plots for the effect of phospholipid molar ratio (X1),stearylamine molar ratio (X2), and sonication time (X3) on PS.

Caff/liposomes entrapment efficiency ranged from 94.35 ± 1.9 to 84.97 ± 1.1%, as shown in Fig. 1A-B. The ANOVA analysis revealed that the phospholipid molar ratio significantly affected the EE% of caffeine in the liposomal formulations. However, boosting the molar ratio of stearylamine led to a slightly insignificant increase in the EE%. Lengthening the sonication time led to a minor increase in the EE% values. The next equation is the polynomial model for EE% (Y_1_):${\mathrm{EE}}\% = + {\mathrm{89}}.{\mathrm{22}} + {\mathrm{4}}.{\mathrm{35A}} + 0.{\mathrm{33B}} + 0.{\mathrm{39C}} + 0.{\mathrm{43AB}} - {\mathrm{1}}.{\mathrm{46AC}} - 0.{\mathrm{55BC}}$

#### Particle size

It is one of the most crucial properties of liposomes, influencing their physical properties and in vivo performance as drug delivery carriers. Therefore, to obtain a liposomal formula with the desired size, the formula was fabricated at various molar ratios of phospholipid to cationic surfactant, and the size and distribution were subsequently examined. Mean PS was determined for all formulations and ranged from 179.73 ± 3.1 to 1598 ± 12.4 nm. The PS results were analyzed using the Box-Cox power transformation to meet the assumptions of normality and homoscedasticity required for regression analysis. The Box-Cox plot indicated the need for transformation, with an optimal lambda value of −0.5. Accordingly, an inverse-square transformation was applied to the PS data to improve model fit and stabilize variance. This approach enhanced the accuracy and reliability of the predictive model for PS. The results showed that X_2_ significantly influenced the PS of the liposomes, as shown in Fig. 1C-D. Increasing the stearylamine molar ratio led to a subsequent increase in liposomes’ PS, whereas increasing the phospholipid molar ratio led to a slight increase in vesicle size. The sonication time slightly decreased PS. The next equation is the PS (Y_2_) polynomial model:

5 $${\mathrm{PS}} = + {\mathrm{735}}.{\mathrm{91}} + {\mathrm{5}}0.0{\mathrm{87A}} + {\mathrm{644}}.{\mathrm{712B}} - {\mathrm{7}}.{\mathrm{52C}} + {\mathrm{167}}.{\mathrm{28AB}} + {\mathrm{41}}0.{\mathrm{89AC}} + {\mathrm{4}}.0{\mathrm{25BC}}$$

#### Polydispersity index

**Fig. 2:**
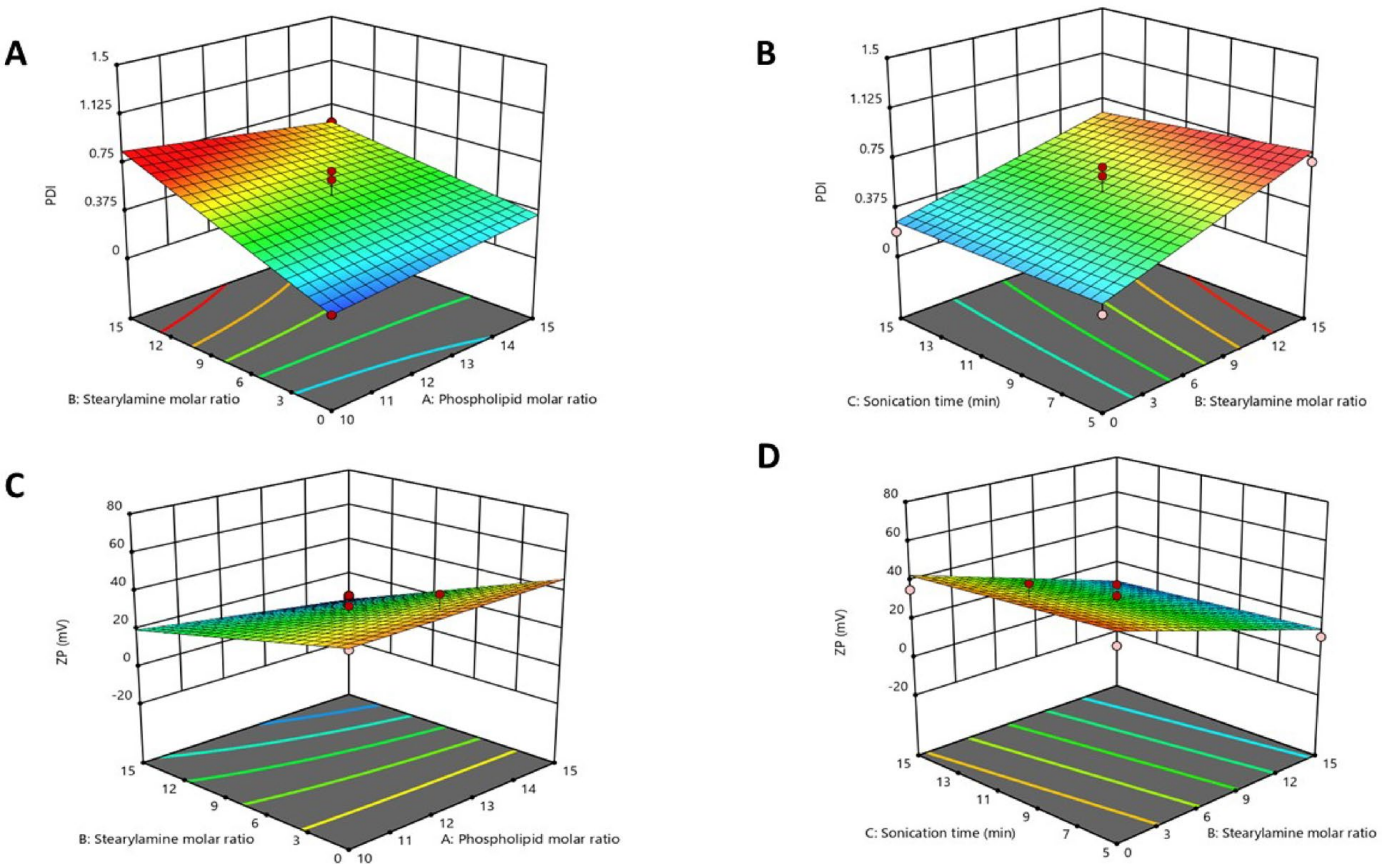
(A-B). Three–dimensional response surface plots for the effect of phospholipid molar ratio (X1), stearylamine molar ratio (X2), and sonication time (X3) on PDI. (C-D). Three–dimensional response surface plots for the effect of phospholipid molar ratio (X1), stearylamine molar ratio (X2), and sonication time (X3) on ZP.

PDI values of all formulations represented a wide range from 0.19 ± 0.02 to 0.73 ± 0.04, as illustrated in Fig. 2A-B. Increasing the phospholipid molar ratio had a negligible effect on PDI values. On the contrary, increasing the stearylamine molar ratio had a positive impact on the PDI values, whereas increasing the sonication time slightly decreased the PDI values. The next equation is the polynomial model for PDI%(Y_3_):${\mathrm{PDI}} = + 0.{\mathrm{5}}0 - 0.0{\mathrm{24A}} + 0.{\mathrm{22B}} - 0.0{\mathrm{45C}} - 0.0{\mathrm{93AB}} + 0.0{\mathrm{43AC}} - 0.0{\mathrm{36BC}}$

#### Zeta potential

ZP is an important indicator of nanocarrier stability^40^. The ZP values of the prepared formulations varied from − 51 ± 1.2 to −4.66 ± 0.8mV, as shown in Fig. 2C-D. The surface charge of liposomes was significantly affected by the molar ratio of stearylamine; however, the inclusion of a high molar ratio of the cationic surfactant into the liposomal bilayer did not convert the negative charge to a positive one; instead, only a reduction in the negative charge intensity was observed. The sonication time slightly decreased the ZP values. The next equation is the polynomial model for ZP (Y_4_):

6 $${\text{ZP }} = + {\mathrm{28}}.{\mathrm{68}} - {\mathrm{2}}.0{\mathrm{1A}} - {\mathrm{17}}.0{\mathrm{8B}} - {\mathrm{3}}.{\mathrm{11C}} - {\mathrm{3}}.{\mathrm{88AB}} - {\mathrm{3}}.{\mathrm{7}}0{\mathrm{AC}} - 0.0{\mathrm{6}}0{\mathrm{BC}}$$

### Selection of the optimized preparation

**Fig. 3:**
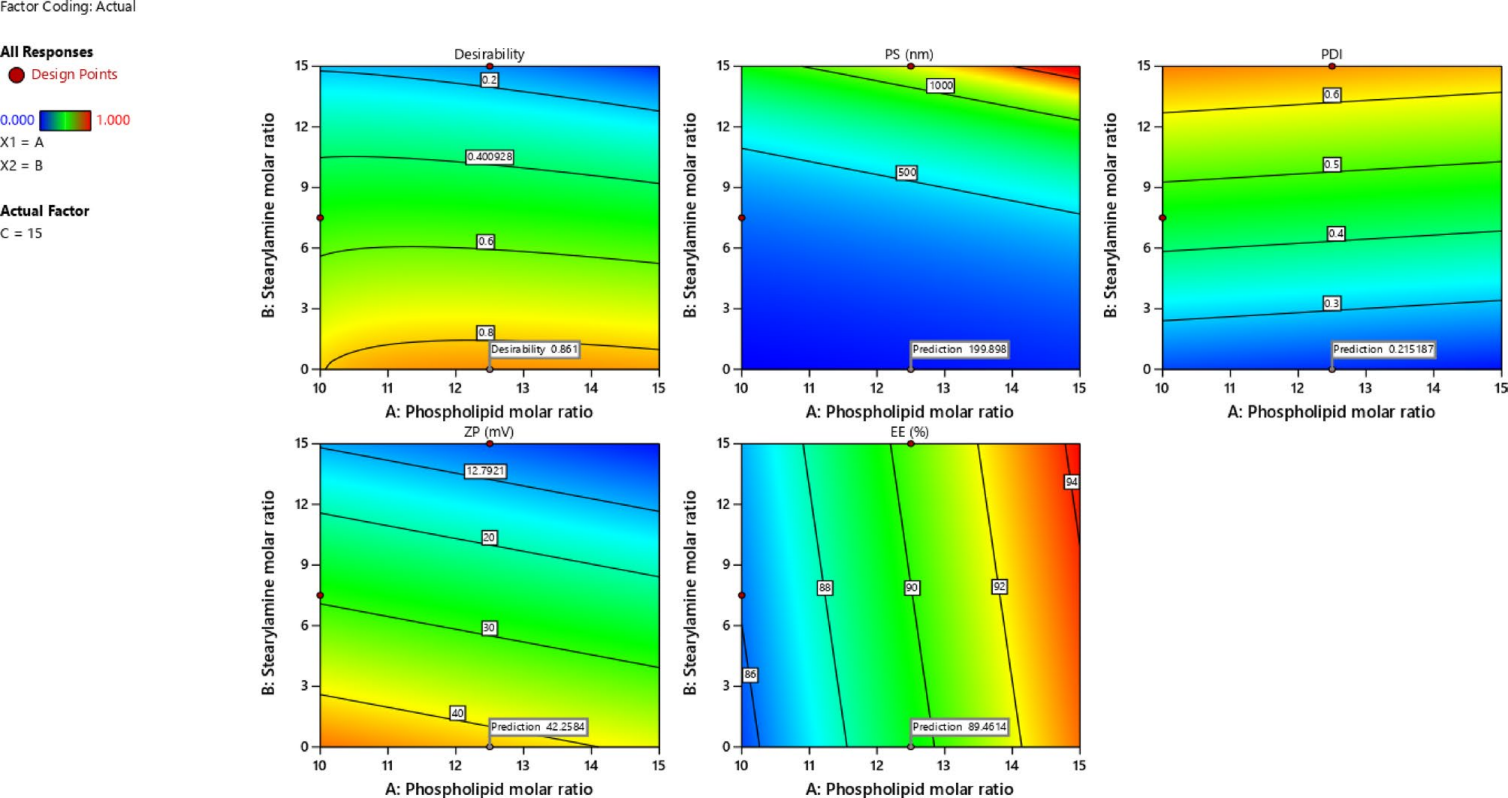
Contour plots for the optimization parameters.

The required constraints were to maximize EE% and ZP, while minimizing PS and PDI. The software, with a desirability value of 0.861, suggested preparation (P5). The contour plots for the optimization parameters are presented in Fig. 3.

### In vitro drug release study

**Fig. 4:**
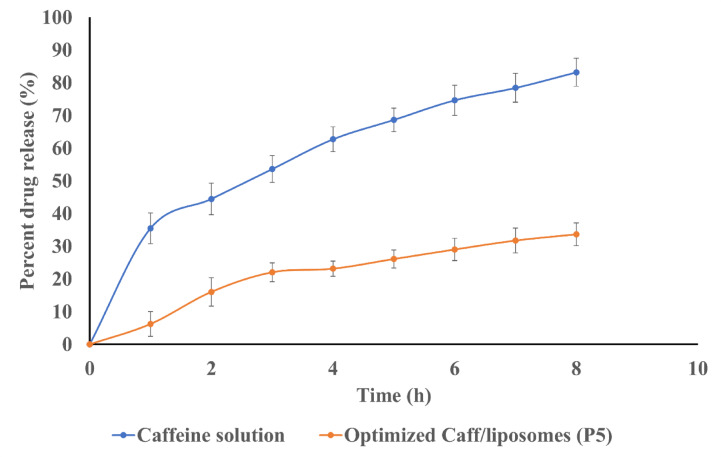
In vitro drug release profile of caffeine solution and optimized Caff/liposomes formula (P5).

The in vitro release profile of caffeine from the optimized liposomal formula (P5) exhibited sustained release, as shown in Fig. 4, with a Q8% of 33.68 ± 3.5%, compared to 83.21 ± 4.3% achieved by the caffeine solution. The in vitro drug release profiles followed the Higuchi model. The high r^2^ value of the Higuchi model suggests that drug release from liposomal formulation follows a diffusion process, with an initial burst release followed by a more sustained release phase.

### Determination of loading efficiency of SPION

The loading efficiency of SPION was 6.06 µmole per mmole lipid, while the number of SPION encapsulated in each liposome was found to be 3.9 (~ 4).

### Fourier-transform IR spectroscopy

**Fig. 5:**
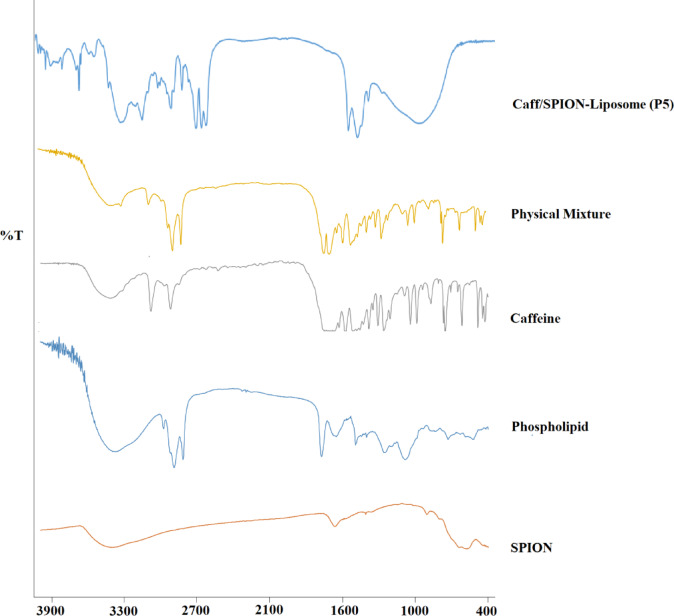
FTIR spectra of the physical mixture of drug and SPION, caffeine, SPION, phospholipid, and P5 (Caff/SPION-liposomes).

FTIR was used to confirm the inclusion of caffeine and SPION within the liposomal vesicles. As presented in Fig. 5, the FTIR spectrum of caffeine typically shows the characteristic peak of the aromatic N-H stretching at 3435 cm^− 1^ and the stretching of H-C at 2953.02 & 3111.18 cm^− 1^. Furthermore, the stretching of the C = O of the amide group appeared at 1651 cm^− 1^, the stretching of C = C at 1598 cm^− 1^, and the stretching of C-N at 1325 cm^− 1^. Similarly, the peak at 1596 cm^–1^ falls in the stretching vibration region of the C = N bond of caffeine, and ring stretching of N = C appeared at 1661.29 cm^− 1^. A strong peak at 1218 cm^–1^ may be due to the combined contribution of stretching vibrations of C = O and C-N bonds of caffeine^41^. The FTIR spectrum of SPION typically exhibited a strong band in the low-frequency region (1000–500 cm^− 1^) due to the iron oxide skeleton. The characteristic band of iron oxide at 567 cm^− 1^ revealed that the particles consisted mainly of Fe_3_O_4_. In addition, the peak at 3421 cm^− 1^ is related to the formation of hydroxyl groups on the surface of SPION.

### Characterization of BM-MSCs

**Fig. 6:**
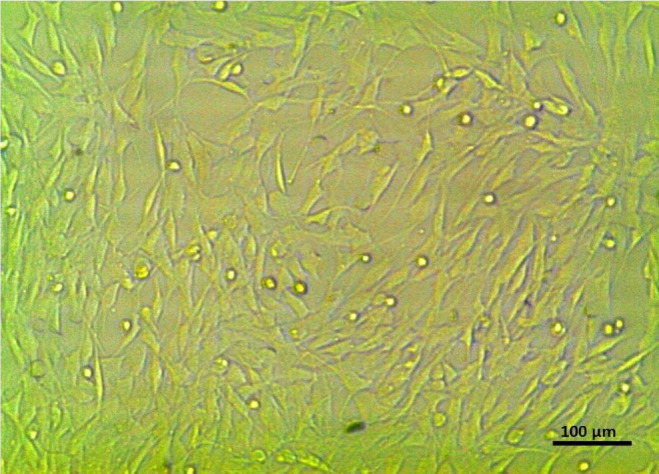
Spindle-shaped morphology of isolated BM-MSCs.

**Fig. 7:**
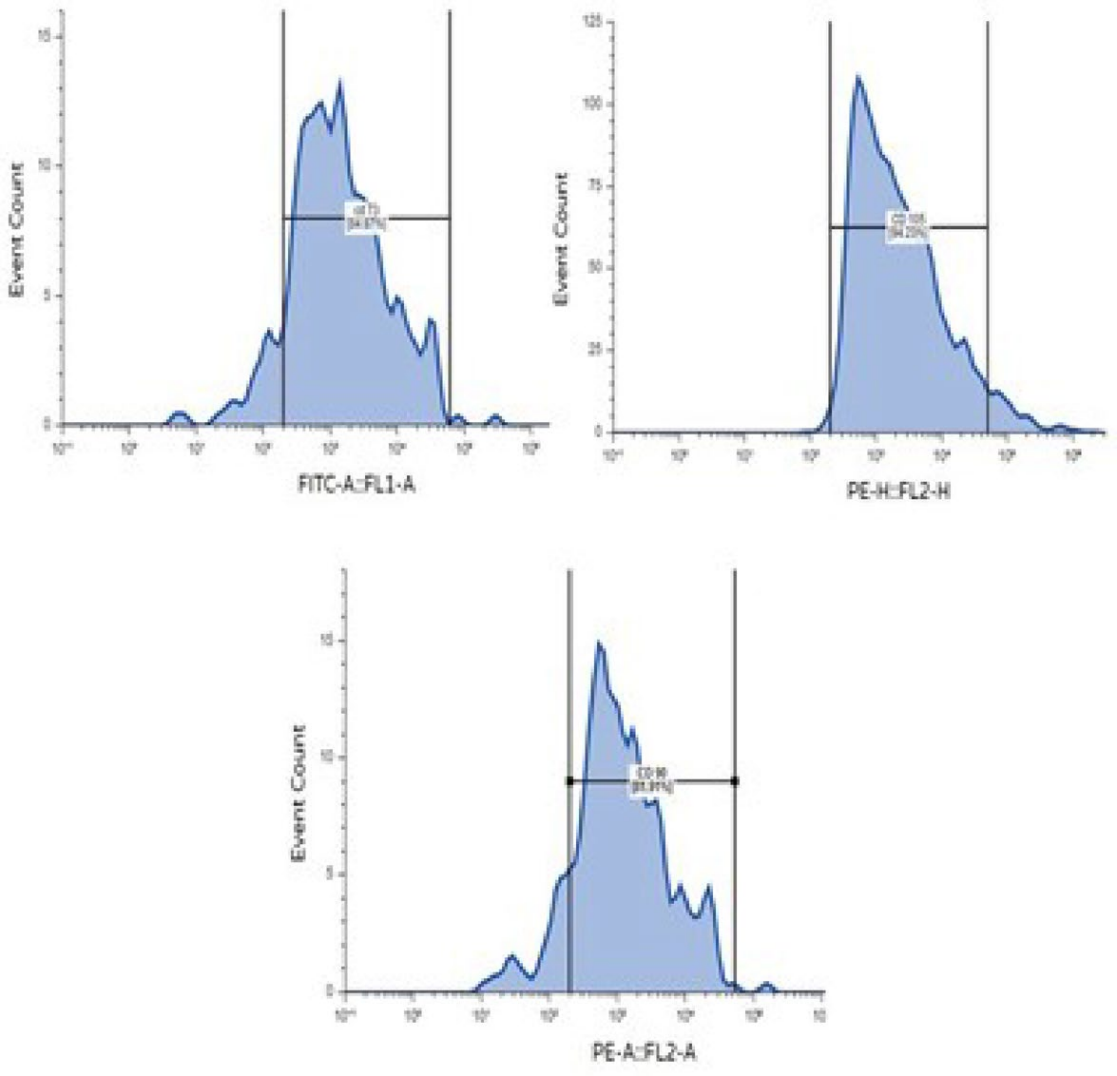
Flowcytometric analysis of isolated BM-MSCs against CD 73, CD 105, and CD 90 showing positive reaction 84.87%, 94.23%, and 81.90% respectively.

Bone marrow MSCs (BM-MSCs) were successfully isolated. They exhibited a spindle-shaped morphology under the inverted microscope, as shown in Fig. 6. The flow cytometric analysis showed positive reactions against MSCs CD markers 73, 105, and 90, with rates of 84.87%, 94.23%, and 81.90%, respectively, as shown in Fig. 7.

### Characterization of exosomes

Successful isolation of exosomes was achieved, and the BCA protein concentration was adjusted to 200 µg/mL.

**Fig. 8:**
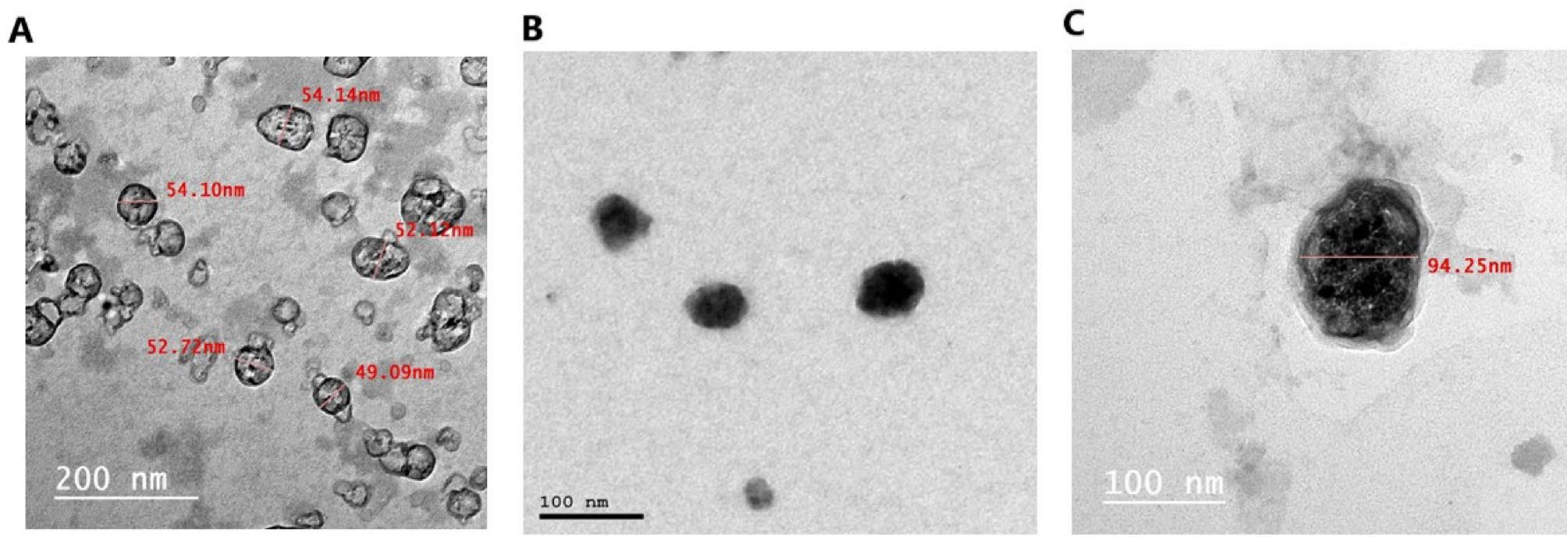
Transmission electron microscopy (TEM) characterization of nanocarrier components and hybrid system. TEM images showing morphological analysis of: (a) Exosomes - intact circular vesicles with average diameter of 49.09-54.14 nm displaying characteristic bilayer membrane structure with no signs of rupture or damage; (b) Caff/SPION liposomes (P5)- spherical vesicles exhibiting dark core (indicating SPION presence) surrounded by light lipid bilayer border, with observed particle size of approximately 64.5 nm; (c) Exosome-Caff/SPION liposome hybrid (F1)- larger composite particles (~100 nm diameter) demonstrating successful encapsulation of caffeine/SPION liposomes within exosome carriers. Samples were prepared on copper grids, stained with phosphotungstic acid for 10 minutes, and imaged using Jeol T300 TEM system at 20-30 kV accelerating voltage.

TEM image of exosomes, as presented in Fig. 8A, revealed normal circular morphology with an average diameter size of 49.09–54.14 nm, with no signs of ruptured or broken exosomes observed. TEM image of the selected Caff/SPION-liposome preparation (P5) illustrated a spherical vesicle with a dark core and light border, as shown in Fig. 8B. The observed PS was approximately 64.5 nm, which was smaller than the results obtained from the DLS measurement. The exosome-liposome hybrid was larger, in the range of 100 nm, as shown in Fig. 8C. According to DLS measurement, the PS of exosome-liposome hybrid was 152.0 ± 13.5 nm. The EE% of caffeine in exosomal formulae was determined to be 99.04 ± 5.5% for F1 and 88.37 ± 2.3% for F2.

After 7 months of storage at 4 °C, the particles maintained their characteristics as demonstrated in Table 5, indicating the physical stability of the developed system.

**Table 5:** Impact of storage for 7 months at 4 °C on the characteristics of exosome-Caff/SPION liposome hybrid.

Paramter	Before storage	After storage
EE (%)	99.04 ± 5.5	87.65 ± 4.5
PS (nm)	152.0 ± 13.5	177.67 ± 11.0
PDI	0.318 ± 0.09	0.452 ± 0.06
ZP (mV)	−21.5 ± 4.79	−22.5 ± 4.17

*EE* Entrapment efficiency, *PS* Particle size, *PDI* Polydispersity index, *ZP* Zeta potential.

### Biological activity of exosome-Caff/SPION liposome hybrid

#### Effect of exosomes-Caff/SPION liposomes hybrid on serum liver enzymes in TAA-induced liver fibrosis in rats

**Fig. 9:**
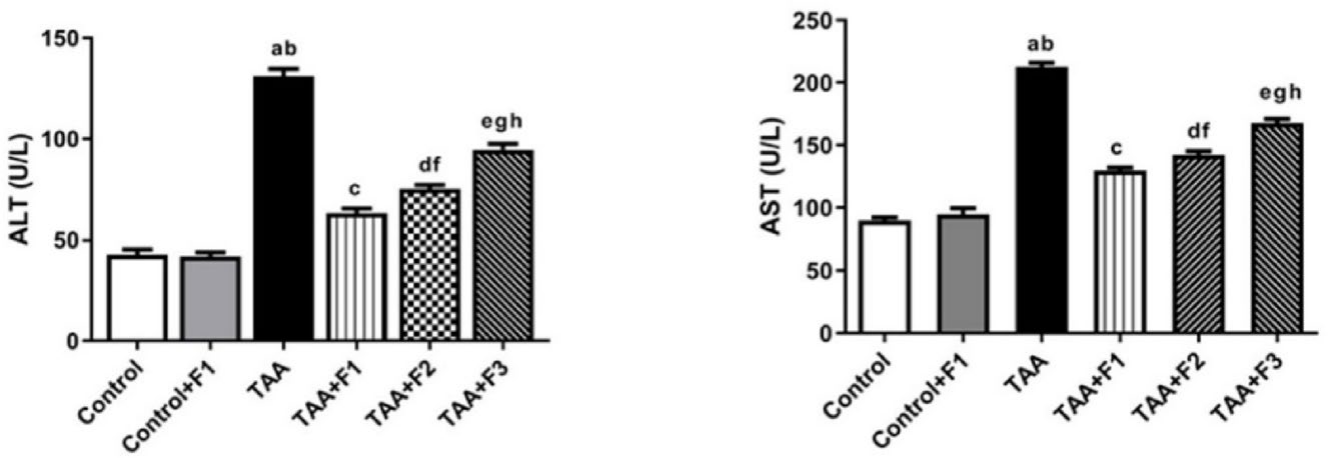
Effect of exosome-Caff/SPION liposome hybrid on liver enzymes in TAA-induced liver fibrosis in rats. a: Significant difference between control group and TAA group. b: Significant difference between control+F1 group and TAA group. c: Significant difference between TAA group and TAA+F1 group. d: Significant difference between TAA group and TAA+F2 group. e: Significant difference between TAA group and TAA+F3 group. f: Significant difference between TAA+F1 group and TAA+F2 group. g: Significant difference between TAA+F1 group and TAA+F3 group. h: Significant difference between TAA+F2 group and TAA+F3 group.

Liver function was estimated by evaluating serum ALT and AST activity. Compared to those in the control and control + F1 groups, the activity of ALT and AST in the TAA group was significantly (*P* < 0.05) greater. In contrast, ALT and AST activity significantly (*P* < 0.05) decreased in the TAA + F1, TAA + F2, and TAA + F3 groups compared to the TAA group. Moreover, ALT and AST activity in the TAA + F1 group was significantly (*P* < 0.05) lower than that in the TAA + F2 and TAA + F3 groups. Notably, the decrease in the activity of serum liver enzymes was more significant (*P* < 0.05) in the TAA + F2 group than in the TAA + F3 group, as shown in Fig. 9.

#### Effect of exosomes-Caff/SPION liposomes hybrid on inflammatory mediators in TAA-induced liver fibrosis in rats

**Fig. 10:**
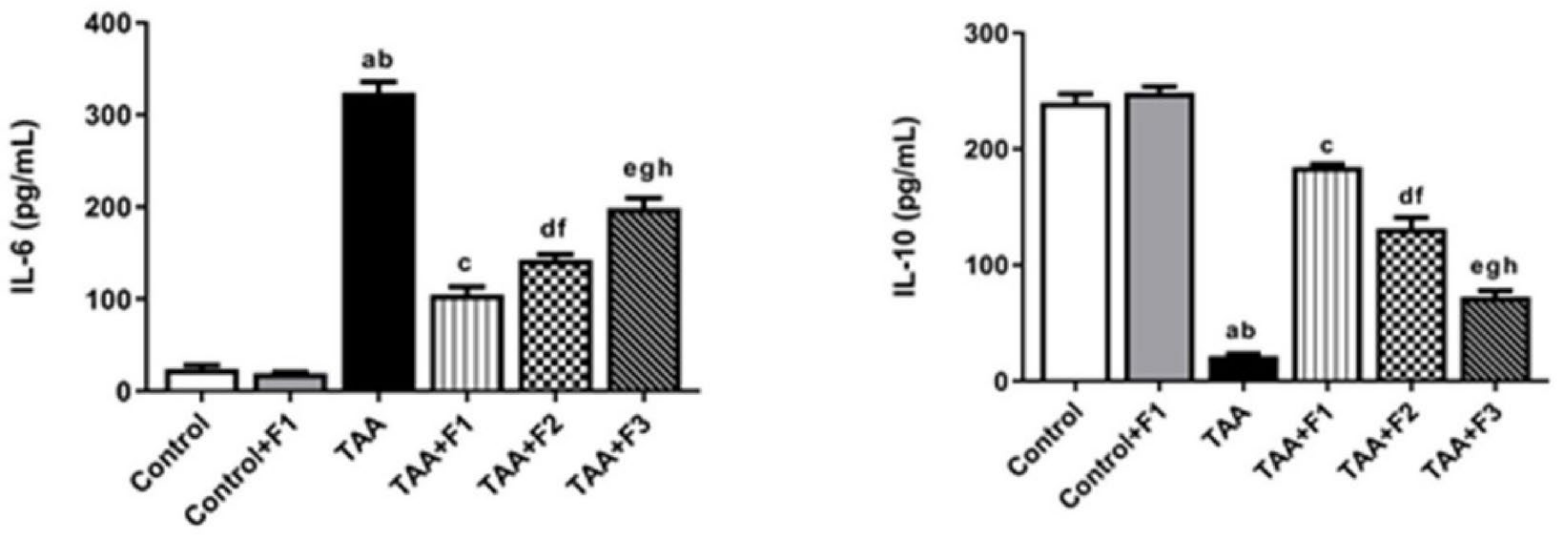
Effect of exosome-Caff/SPION liposome hybrid on inflammatory mediators in TAA-induced liver fibrosis in rats. a: Significant difference between control group and TAA group. b: Significant difference between control+F1 group and TAA group. c: Significant difference between TAA group and TAA+F1 group. d: Significant difference between TAA group and TAA+F2 group. e: Significant difference between TAA group and TAA+F3 group. f: Significant difference between TAA+F1 group and TAA+F2 group. g: Significant difference between TAA+F1 group and TAA+F3 group. h: Significant difference between TAA+F2 group and TAA+F3 group.

The results in Fig. 10 indicated a significant (*P* < 0.05) enhancement in the serum level of the proinflammatory mediator (IL-6) and a significant decrease in the anti-inflammatory one (IL-10) upon TAA injection versus the control and control + F1 groups. On the other hand, treatment with F1 significantly (*P* < 0.05) improved the inflammatory status compared to the TAA group. The improvement in inflammatory status was more pronounced (*P* < 0.05) in the TAA + F1 group than in the TAA + F2 and TAA + F3 groups. Additionally, the improvement was significantly better (*P* < 0.05) in the TAA + F2 group than in the TAA + F3 group.

#### Effect of exosomes-Caff/SPION liposomes hybrid on NF-κB/Myd88/TLR-4 pathway in TAA-induced liver fibrosis in rats

**Fig. 11:**
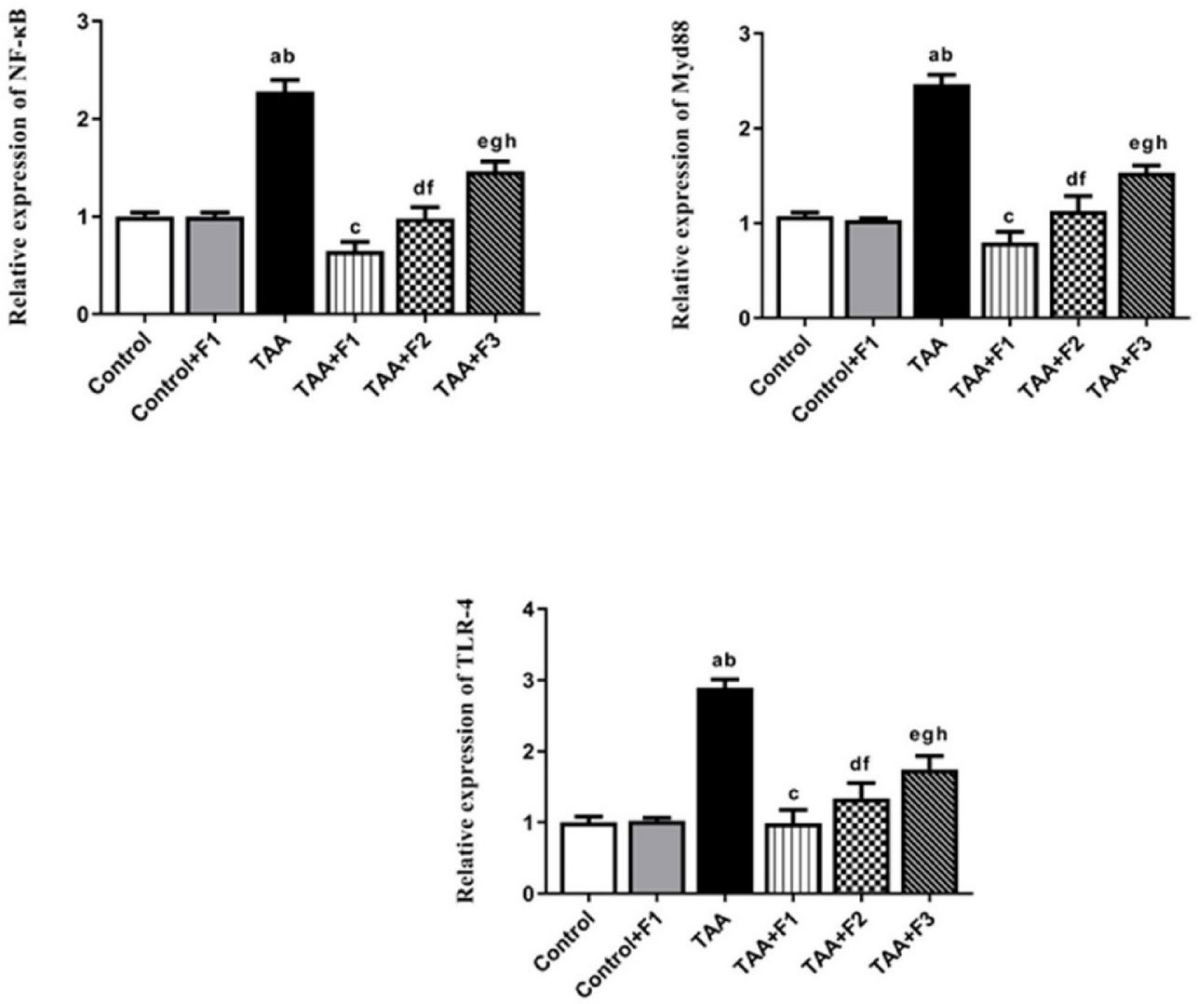
Effect of exosome-Caff/SPION liposome hybrid on NF-κB/ Myd88/ TLR-4 pathway in TAA-induced liver fibrosis in rats. a: Significant difference between control group and TAA group. b: Significant difference between control+F1 group and TAA group. c: Significant difference between TAA group and TAA+F1 group. d: Significant difference between TAA group and TAA+F2 group. e: Significant difference between TAA group and TAA+F3 group. f: Significant difference between TAA+F1 group and TAA+F2 group. g: Significant difference between TAA+F1 group and TAA+F3 group. h: Significant difference between TAA+F2 group and TAA+F3 group.

On the molecular level, the NF-κB/Myd88/TLR-4 pathway was significantly (*P* < 0.05) upregulated in hepatic tissues of the TAA group compared to the control and control + F1 groups. On the contrary, it was significantly downregulated (*P* < 0.05) in the TAA + F1, TAA + F2, and TAA + F3 groups compared to the TAA group. The downregulation of this pathway was more observed (*P* < 0.05) in the TAA + F1 group than in the TAA + F2 and TAA + F3 groups. Moreover, the downregulation was significantly greater (*P* < 0.05) in the TAA + F2 group than in the TAA + F3 group, as shown in Fig. 11.

#### Effect of exosomes-Caff/SPION liposomes hybrid on hepatocyte fibrosis in TAA-induced liver fibrosis in rats

**Fig. 12:**
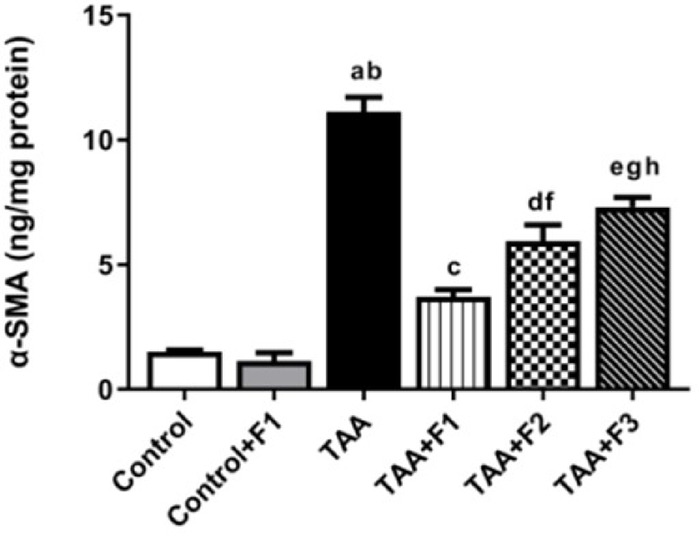
Effect of exosome-Caff/SPION liposome hybrid on hepatic α-SMA protein in TAA-induced liver fibrosis in rats. a: Significant difference between control group and TAA group. b: Significant difference between control+F1 group and TAA group. c: Significant difference between TAA group and TAA+F1 group. d: Significant difference between TAA group and TAA+F2 group. e: Significant difference between TAA group and TAA+F3 group. f: Significant difference between TAA+F1 group and TAA+F2 group. g: Significant difference between TAA+F1 group and TAA+F3 group. h: Significant difference between TAA+F2 group and TAA+F3 group.

Rats with TAA-induced fibrosis showed higher hepatic α-SMA protein levels (*P* < 0.05) compared to the control and control + F1 groups. F1 treatment significantly decreased (*P* < 0.05) the α-SMA protein level in the hepatic tissues compared to the TAA group. The reduction of hepatic α-SMA protein level was more pronounced (*P* < 0.05) in the TAA + F1 group than in the TAA + F2 and TAA + F3 groups. It is worth noting that hepatic α-SMA protein level was significantly (*P* < 0.05) reduced in the TAA + F2 group compared to the TAA + F3 group, as shown in Fig. 12.

#### Effect of exosomes-Caff/SPION liposomes hybrid on hepatocyte proliferation in TAA-induced liver fibrosis in rats

**Fig. 13:**
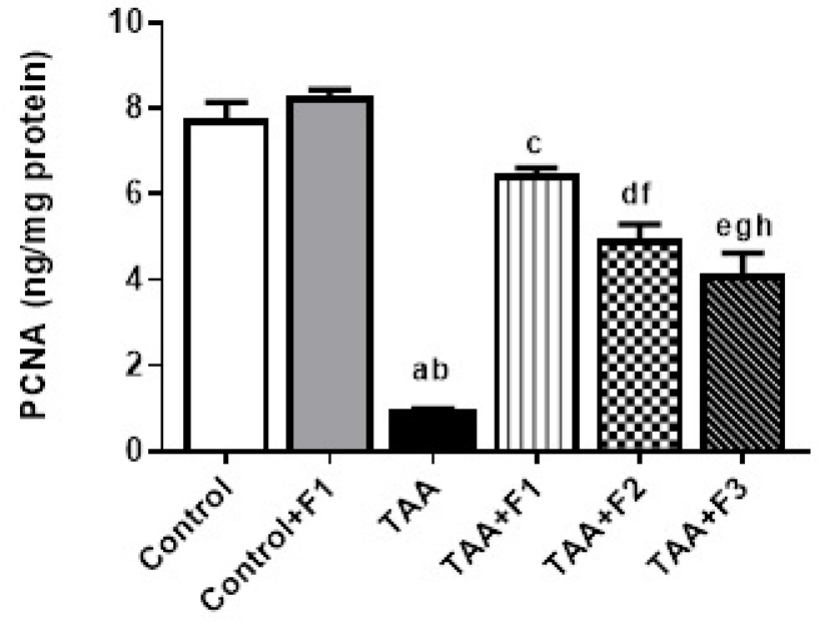
Effect of exosome-Caff/SPION liposome hybrid on hepatic PCNA protein in TAA-induced liver fibrosis in rats. a: Significant difference between control group and TAA group. b: Significant difference between control+F1 group and TAA group. c: Significant difference between TAA group and TAA+F1 group. d: Significant difference between TAA group and TAA+F2 group. e: Significant difference between TAA group and TAA+F3 group. f: Significant difference between TAA+F1 group and TAA+F2 group. g: Significant difference between TAA+F1 group and TAA+F3 group. h: Significant difference between TAA+F2 group and TAA+F3 group.

Notably, PCNA, which is the basic element in DNA replication and repair, was significantly lower (*P* < 0.05) in the TAA group, contrary to the control and control + F1 groups. On the other hand, hepatic PCNA protein content was significantly (*P* < 0.05) elevated in the TAA + F1, TAA + F2, and TAA + F3 groups compared with the TAA group. The enhancement of PCNA protein was more significant (*P* < 0.05) in the TAA + F1 group than in the other treated groups. Additionally, the hepatic PCNA protein level was significantly higher (*P* < 0.05) in the TAA + F2 group compared to the TAA + F3 group, as shown in Fig. 13.

#### Effect of exosomes-Caff/SPION liposomes hybrid on the histopathological changes in TAA-induced liver fibrosis in rats

**Fig. 14:**
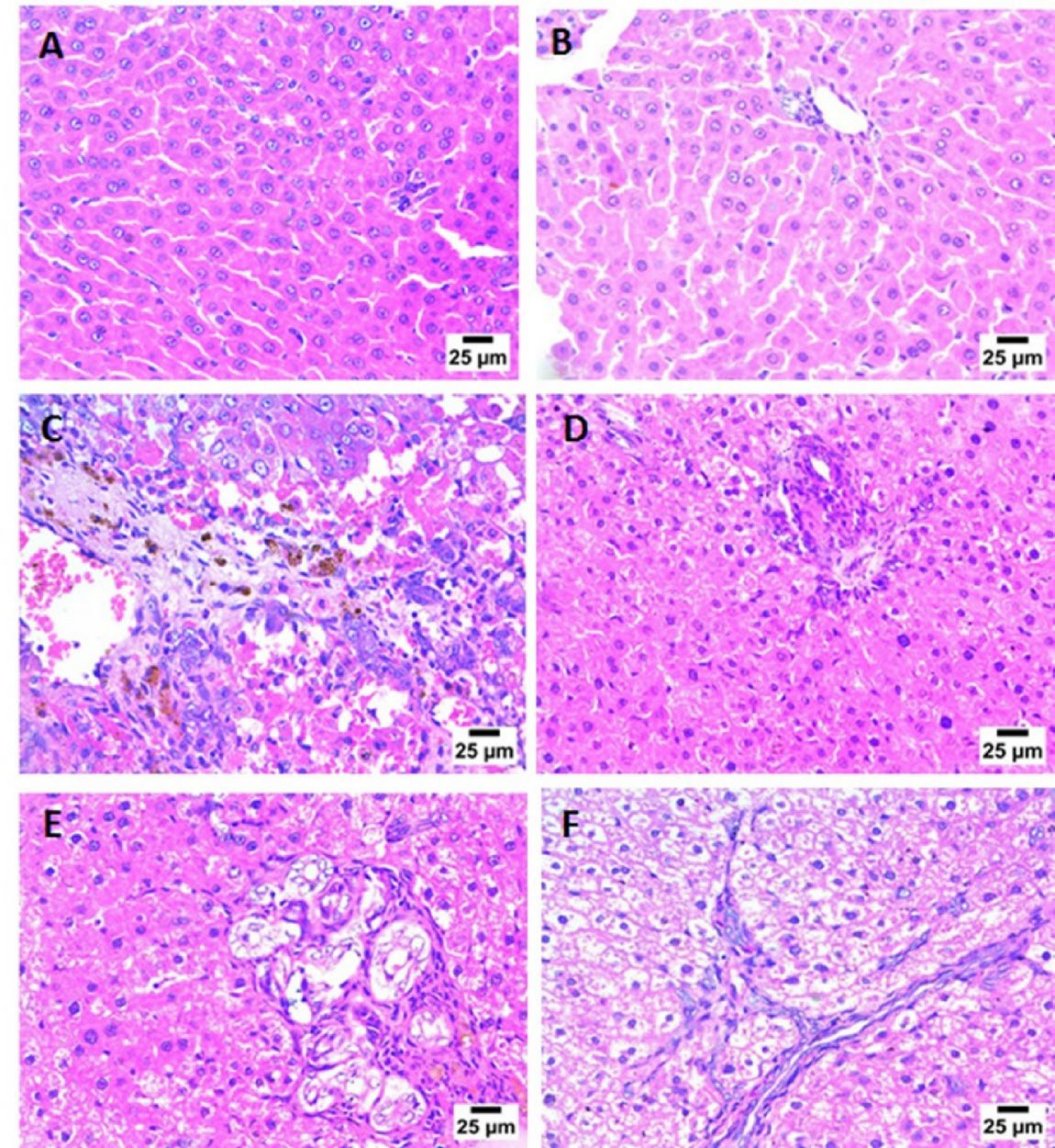
H&E-stained sections of hepatic tissues in the different studied groups ( ×400, scale bar: 25 μm). A: control group. B: control+F1 group, C:TAA group, D: TAA+F1 group, E: TAA+F2 group, F: TAA+F3 group.

H&E-stained liver sections from the control group, as shown in Fig. 14A, and the control + F1 group (Fig. 14B), showed normal histological structure of hepatocytes and portal area, indicating the safety of the developed system on healthy liver cells. In contrast, HE-stained liver sections from the hepatic fibrosis (TAA) group showed severe liver fibrosis, characterized by the formation of fibrous connective tissue septa with haemorrhage, infiltration by mononuclear inflammatory cells, and bile ductules (Fig. 14C). The TAA + F1 group exhibited infiltration of mononuclear inflammatory cells, the presence of severe nuclear pyknosis, and mild portal fibrosis in some hepatocytes (Fig. 14D). The TAA + F2 group demonstrated portal fibrosis with mononuclear inflammatory cell infiltration, a moderate number of newly formed bile ductules, and some hepatocytes showing necrobiotic changes, including severe nuclear pyknosis and vacuolar degeneration (Fig. 14E). The TAA + F3 group showed the formation of thin, long fibrous connective tissue septa, with newly formed bile ductules and hepatocytes exhibiting necrobiotic changes, including severe nuclear pyknosis and vacuolar degeneration (Fig. 14F).

#### Toxicity study

The potential toxicity of the F1 hybrid was further assessed in the kidney and spleen tissues of healthy rats in Group 2 through histopathological examination. The results revealed no signs of toxicity, with both organs exhibiting normal histological architecture. (Data available upon request).

## Discussion

The current work aimed to develop an engineered liposome-exosome hybrid loaded with caffeine to prohibit liver fibrosis induced in rats. For improved targeting and homing of the engineered liposome-exosome hybrid to the liver, the nanocarriers were loaded with SPION.

The caffeine-loaded liposome formulations were successfully developed using the thin-film hydration method. It was found that the molar ratios of phospholipids and stearylamine influenced the liposomes’ characteristics. The EE% was enhanced by increasing the lipid molar ratio (X_1_), probably due to the formation of more tightly packed vesicles capable of encapsulating hydrophilic drugs and minimising their leakage^42^. Moreover, the effect of increasing the phospholipid molar ratio on EE% was dependent on the sonication time, with the effect being more pronounced at shorter sonication durations.

Additionally, increasing the stearylamine molar ratio (X_2_) increased the EE%, as the cationic surfactant most likely interacts electrostatically with the negatively charged phospholipids in the liposome bilayer, thereby enhancing liposome stability and retaining the encapsulated drugs^43^. Increasing the stearylamine molar ratio also led to a subsequent increase in the PS of the liposomes, as previous studies confirmed the shape conversion of stearylamine-based liposomes from multilamellar vesicles (MLVs) to large unilamellar vesicles (LUVs)^44^.

Additionally, a high concentration of stearylamine within the vesicle increased the EE% of caffeine, thereby increasing PS. At the same time, increasing the molar ratio of phospholipids led to a slight increase in the PS, as increasing the lipid component in the vesicles will subsequently result in increased vesicular size^45^. The effect of phospholipid molar ratio on PS varied with sonication time: increasing the phospholipid molar ratio increased PS at long sonication times.

The sonication time was slightly decreased, which likely resulted in a decrease in PS, probably due to the increased breaking of aggregated vesicles^46^. Jain et al. (2012) postulated that high energy input to the nanoformulations would destabilise them via mechanical collision and partially due to the heat generated in the microenvironment^47^.

Increasing the phospholipid molar ratio had a negligible effect on PDI values. On the contrary, increasing the stearylamine molar ratio had a positive impact on PDI values. This is because stearylamine reduces the intensity of the negative charge on the liposomes through neutralisation, which may enhance vesicle aggregation. Still, it was insufficient to cause a charge inversion due to a possible asymmetrical distribution of stearylamine in the bilayer^48^.

Although the phospholipid molar ratio and sonication times had no significant impact on ZP (p-values 0.5216 and 0.501, respectively), it was found that increasing the phospholipid molar ratio enhances ZP at shorter sonication.

Stearylamine was included in the initial formulation design as a cationic surfactant commonly used to modify the surface charge of liposomes, which may enhance encapsulation efficiency, colloidal stability, and cellular interactions. The amount of stearylamine used in liposome formulations is typically reported within a range of about 5 to 15 mol% relative to the total lipid content^43^. However, in the current study, the impact of streaylamine molar ratio on the characteristics of the liposomal was extended from 0 to about 50 mol% of total lipid content, where amounts beyond may lead to stability or toxicity concerns, although this, the complete charge inversion to a positive one was not attained even in the liposomal formula comprising a higher content of stearylamine. These results indicated that the conversion of liposome surface charge might not be a linear function of stearylamine concentration. Therefore, the optimized formula consisted of phospholipid without stearylamine.

The optimized caffeine-loaded liposomes (P5) released caffeine significantly more slowly than the caffeine solution, demonstrating effective encapsulation and controlled release behaviour. Such a profile supports the intended design of the liposomal system for targeted and sustained drug delivery. Additionally, controlled release from liposomes may enhance drug accumulation at the target site, improve efficacy, and reduce systemic exposure^49^.

FTIR results showed that the caffeine bands were preserved in the physical mixture spectrum, indicating no interference between the drug and other excipients. Hydrogen bonding between lecithin, caffeine, and SPION may be represented by the disappearance of the aromatic N-H and H-C stretching of the caffeine bands in the P5 spectrum, as well as the disappearance of the SPION bands. This could suggest that the caffeine and SPIONs are effectively encapsulated within the liposomal bilayers.

The TEM micrograph showed smaller PS than those obtained using DLS. In DLS, the solvation layer and any surface-bound molecules affect the hydrodynamic diameter estimation. The hydration layer is not observed with TEM, and only the projected area of the liposome’s core is observed^50^.

The optimized formula (P5) was further encapsulated into exosomes to generate the exosome-Caff/SPION liposome hybrid, which was evaluated for its efficacy in improving liver function, mitigating inflammatory status, alleviating liver fibrosis, and promoting hepatocyte proliferation in rats with TAA-induced liver fibrosis.

The successful isolation of BM-MSCs is a pivotal step in regenerative medicine and cellular therapies. The spindle-shaped morphology is a characteristic of MSCs, indicating that the isolation process was effective. Furthermore, the flow cytometric analysis demonstrated a high percentage of positive reactions for MSCs-specific surface markers: CD73 (84.87%), CD105 (94.23%), and CD90 (81.90%). These results confirm that the isolated cells possess the essential stem cell properties necessary for further applications, aligning with established criteria for MSC identification^26^.

The successful isolation of exosomes from BM-MSCs represents a significant advancement in understanding intercellular communication and potential therapeutic applications. The BCA protein concentration of the exosomes was standardised to 200 µg/mL, ensuring consistency for subsequent analyses. TEM images revealed that the exosomes maintained a normal circular morphology, with an average diameter of 49.09–54.14 nm, consistent with the reported sizes of exosomes. The absence of ruptured or broken exosomes suggests that the isolation process preserved their integrity, which is crucial for their functional efficacy^51^.

Loading exosomes with P5 to generate an exosome-Caff/SPION liposome hybrid (F1) resulted in dark-colored exosomes with a granular appearance on TEM. The average diameter of these loaded exosomes was about 100 nm. This increase indicates a successful encapsulation of the liposomes within the exosomes.

Regarding the biological experiment, the induction of liver fibrosis was accomplished using TAA, a well-established model for liver fibrosis^52^. TAA, a thiono-sulfur-containing compound (CH_3_CSNH_2_), goes through an immense metabolism to produce thioacetamide-S-oxide (TAA-S-oxide) as well as acetamide (CH_3_CONH_2_)^53^. After that, TAA-S-oxide is further metabolized by cytochrome P-450 monooxygenases to produce TAA-S-dioxide, which exerts toxicity *via* binding to macromolecules of hepatocytes and induces centrilobular necrosis due to ROS production^54^. The yielded ROS causes a variety of pathophysiological events by advancing lipid peroxidation in the cell membrane, leading to cellular injury and the leakage of liver enzymes into the serum. Comparable to the results obtained, TAA-induced hepatotoxicity was documented by heightened liver enzymes in serum as well as histological disfiguration of liver tissue^55^.

Toxins generated from the metabolism of TAA also target liver cells, inducing hepatocyte dysfunction and inflammation, as well as cytokine release^56^. This was observed in the study through the increase in proinflammatory cytokine (IL-6) and the decrease in anti-inflammatory cytokine (IL-10). Additionally, the NF-κB/Myd88/TLR-4 pathway was upregulated. Persistent inflammatory factors stimulate hepatic stellate cells (HSCs) to secrete fibrotic factors, which enhance extracellular matrix (ECM) formation and ultimately lead to liver fibrosis^57^. HSCs and fibrotic tissue activation are regulated by inflammatory cytokines as well as ROS^58^. HSC activation results in the elevated expression of fibrotic biomarkers, such as α-SMA^59^, via the TGF-β1/Smad signaling pathway^60^. PCNA functions as an adjunct protein to DNA polymerase. PCNA expression is upregulated in the S phase of mitosis compared to DNA replication^61^. In the current investigation, it was observed that TAA-induced liver injury inhibits PCNA. This may be due to the occurrence of chronic inflammation, which is considered a key pathophysiologic mechanism of hepatic fibrosis, where there is an inverse relationship between inflammation and PCNA levels^62^.

Rats with TAA-induced liver injury were subjected to three different treatments: exosome-Caff/SPION liposome hybrid (F1), exosome-Caff/liposome hybrid (F2), and exosome-SPION/liposome hybrid (F3). The biochemical analysis and histopathological study revealed a significant improvement in hepatic injury in all treated groups, in the order of F1 > F2 > F3.

F3-treated rats received solely exosomes, with no additional medical treatment; however, a significant hepatoprotective effect was observed. This finding is likely because exosomes carry bioactive cargo, including proteins, RNA, DNA, lipids, and metabolites^63^. Many studies have indicated that exosomes derived from MSCs can contribute to the therapeutic potential of MSCs by enhancing intercellular communication and delivering paracrine factors, thereby facilitating anti-fibrosis, immunomodulation, and liver regeneration^64^. Exosomes have been shown to mitigate oxidative stress^65^, thereby restoring cell membrane integrity and reducing liver enzyme leakage in the serum. They can attenuate inflammatory reactions by minimising the infiltration of inflammatory cells, such as macrophages, T cells, and NK cells^66^. Additionally, miRNA-146a^67^, miRNA-155^68^, and specific cytokines, such as IL-10, as well as growth factors like hepatocyte growth factor (HGF), participate in MSC-exosome-mediated immunomodulation^69^. This was emphasised in the study by the lowering of the IL-6 protein level, accompanied by the downregulation of NF-κB/Myd88/, and TLR-4 gene expression, and the rise of the IL-10 protein level.

Activated HSCs are considered the primary fibroblasts that have a principal function in the development of liver fibrosis. In normal conditions, HSCs stay dormant. However, when the liver is damaged, HSCs become activated and transformed into cell types associated with fibrosis. They produce collagen, fibronectin, and other substances that promote the formation of collagen fibres, leading to liver fibrosis^70^. Exosomes significantly enhance HSC activation^71^, thereby downregulating the expression of collagen and other fibrosis-related proteins^12^, such as α-SMA. Additionally, by suppressing inflammatory signalling pathways, such as the NF-κB pathway^71^, exosomes can exert an inhibitory effect on fibrosis^72^. Moreover, miRNAs enriched in MSC-exosomes, such as miR-223, miR-10a, and miR-486^73^, are implicated in their antifibrotic mechanisms and are capable of promoting cell proliferation^74^. In a previous study, the administration of MSC-derived exosomes was found to reverse CCl4-induced liver injury in mice, as indicated by the active proliferation of hepatocytes, as evidenced by increased PCNA, cyclin D1, and cyclin E expression^75^.

As indicated by the results, the combination of exosomes and caffeine (F2: exosome-Caff/liposome hybrid) for managing liver fibrosis has proven to be more efficient than F3, despite the absence of SPION in F2, unlike in F3. This finding indicates the effect of caffeine as a hepatoprotective agent. The administration of F2 significantly inhibited the serum levels of liver enzymes. This agrees with Ruhl and Everhart (2005), who stated that the consumption of caffeine (more than 2 cups/day) is associated with a lower risk of high ALT levels and chronic liver disease compared to non-coffee drinkers^76^. Rezaie et al. (2014) demonstrated that caffeine can prevent liver damage by preserving the integrity of the plasma membrane, suppressing liver enzyme leakage through membranes, and exhibiting hepatoprotective activity^77^.

Furthermore, Vargas-Pozada et al. stated that caffeine exerts an anti-inflammatory impact by attenuating TLR-4 and NF-κB protein levels in an experimental NASH rat model^19^. Notably, caffeine attenuates oxidative stress by activating the Nrf-2 signaling pathway^78^. Therefore, attenuation of oxidative stress, with subsequent reduction of ROS, may decrease inflammation and the induction of fibrosis^79^. Caffeine decreases α-SMA protein levels by inhibiting inflammation through the activation of several chemokine-mediated proinflammatory signalling pathways, such as the TGF-β/Smad3 pathway. These pathways, secreted by injured hepatocytes, may be responsible for the increased expression of fibrotic biomarkers, such as α-SMA^58^. Additionally, hepatocyte proliferation was increased upon treatment with caffeine, as the inhibition of inflammation by caffeine led to the stimulation of proliferation, thereby increasing the PCNA protein level, as shown in this study, which revealed an inverse relationship between inflammation and PCNA level^61^.

Moreover, the encapsulation of caffeine in liposomes may have controlled the rate of caffeine release, increasing the bioavailability and stability of caffeine in targeting liver fibrosis, as indicated by the levels of liver enzymes, inflammatory, fibrotic, and proliferative markers^80^. It was found that excessive deposition of extracellular matrix (ECM) resulting from severe fibrosis hinders drug delivery to fibrotic liver tissues through the Kupffer cells^81^. In this regard, a drug-loaded liposome-exosome hybrid delivery system was employed for both fibroblast-specific delivery of an anti-fibrosis agent to liver fibroblasts and enhanced drug-loading^30^.

Among all tested formulae, F1, the exosome-Caff/SPION liposome hybrid, showed the best results in the current study as it revealed the greatest impact on the liver functions (ALT and AST) and structure, inflammatory (IL-6, IL-10, TLR-4, Myd88, NF-κB) molecules, fibrotic (α-SMA) protein and hepatocytes proliferative (PCNA) capacity in liver fibrosis rat model induced by TAA. This finding suggests that loading the formula with SPION can successfully enhance the targeting and homing of the exosome-liposome hybrid to the injured liver. This finding aligns with a study by Colino et al. (2020), which found that SPIONs were used to target hepatic macrophages and improve pathological processes in the liver^82^. The authors stated that SPION could also enhance passive targeting to the liver via uptake by the reticuloendothelial system, especially by liver macrophages (Kupffer cells), after intravenous administration^82^. Moreover, liposomes and exosomes could facilitate this delivery, as both are also efficiently recognized and internalized by liver cells, leading to the successful delivery of the loaded drug to diseased liver tissue.

It is worth noting that the histopathological evaluation of liver, kidney, and spleen tissues from healthy rats treated with F1 hybrid formulation (Exosome–Caffeine/SPION liposome hybrid) provided further evidence supporting the safety of the developed system. No signs of cellular damage, inflammation, or structural abnormalities were observed in any organ, indicating the absence of systemic toxicity following administration. These findings align with the well-documented biocompatibility of the individual components. SPION is metabolized in the liver, where the iron component is either reused for physiological processes or excreted, minimizing long-term toxicity risks^83^. Caffeine has been widely studied and recognized for its favorable safety profiles at therapeutic doses^84^. Alongside the inherent biocompatibility of exosomes and liposomal carriers^85,86^, the preserved normal histological architecture in non-target organs suggests that the hybrid nanoplatform is well-tolerated and does not induce off-target effects. Collectively, these results support the potential of the F1 hybrid system as a safe and effective drug delivery platform for future therapeutic applications.

## Conclusion

This study reveals that unmedicated exosomes have a pronounced effect on tissue regeneration. The exosome-Caff/liposome hybrid combined the hepatoprotective effect of caffeine with the antifibrotic effect of exosomes, resulting in greater efficiency in managing liver fibrosis. Furthermore, the incorporation of caffeine and SPION into liposomes and their subsequent incorporation into exosomes (exosome-Caff/SPION liposome hybrid) improved the levels of liver enzymes, inflammatory status, fibrotic proteins, and hepatocyte proliferation. This finding indicates the great potential of directing the engineered magnetic exosome-liposome hybrid to the target tissue. In conclusion, the developed nanocarrier could be regarded as a promising and innovative drug-delivery system targeting hepatic stellate cells in the fibrotic liver. Despite these encouraging results, this study did not include pharmacokinetic and biodistribution analyses, which are crucial for fully understanding the in vivo behaviour, circulation time, metabolism, and clearance of the exosome-liposome hybrids. Therefore, future studies incorporating detailed pharmacokinetic profiling will be essential to validate the clinical translational potential of this targeted drug-delivery system.

## Supplementary Information

Below is the link to the electronic supplementary material.

Supplementary Material 1

## Data Availability

All data generated or analyzed during this study are included in this published article **.**.

## Declarations

### **Generative AI and AI-assisted technologies in the writing process**

During the preparation of this work, the author(s) did not use any AI and AI-assisted technologies in the writing process.
