# Supplementary material for: Development of engineered magnetic liposome/exosome hybrid as a novel caffeine nanocarrier for restraining liver fibrosis induced in rats
Source: Sci Rep. 2026 Feb 6;16:5349. doi: 10.1038/s41598-025-31169-w (PMC12880976; doi:10.1038/s41598-025-31169-w)
Supplement: Supplementary file 1 — Supplementary Material 1 [file 41598_2025_31169_MOESM1_ESM.docx]

**Supplementary Data**

**Diagnostic plots**

The factorial model validation was performed by comparing predicted values with experimentally observed results. The close agreement between predicted and actual values, with residuals randomly distributed and normally distributed as confirmed by diagnostic plots, supports the model’s validity.

Figure S1: Plot of predicted versus actual EE% values, illustrating the model's ability to accurately predict EE% based on experimental data.

Figure S2: Plot of predicted versus actual 1/Sqrt PS values, illustrating the model's ability to accurately predict 1/Sqrt PS based on experimental data.

Figure S3: Plot of predicted versus actual PDI values, illustrating the model's ability to accurately predict PDI based on experimental data.

Figure S4: Plot of predicted versus actual ZP values, illustrating the model's ability to accurately predict ZP based on experimental data.
